# Isolation, Characterization, and Application of Bacteriophage for *Salmonella* Control in Broiler Chickens

**DOI:** 10.1155/vmi/6502225

**Published:** 2025-05-19

**Authors:** Wisanu Wanasawaeng, Thotsapol Thomrongsuwannakij, Niwat Chansiripornchai

**Affiliations:** ^1^Department of Veterinary Medicine, Faculty of Veterinary Science, Chulalongkorn University, Bangkok 10330, Thailand; ^2^Akkhraratchakumari Veterinary College, Walailak University, Nakhon Si Thammarat 80160, Thailand; ^3^Centre for One Health, Walailak University, Nakhon Si Thammarat 80160, Thailand

**Keywords:** bacteriophages, broiler chickens, livestock industry, *Salmonella*

## Abstract

The present study explores alternatives to antibiotics for poultry farms. The aims of this study were to isolate and characterize bacteriophages for selection of the appropriate phage, reduce *Salmonella* in the gastrointestinal tract of broiler chickens, and observe gut microbiota alterations after bacteriophage treatments. In this study, bacteriophages were isolated from two broiler chicken farms, two poultry processing plants, a goat farm, and a pig farm in the central region of Thailand. Out of the 33 samples analyzed, 25 (75.5%) tested positive for the presence of *Salmonella* bacteriophages. Among the 63 isolates examined, SEpBS-1 was selected for its ability to infect five *Salmonella* serovars: *S. Enteritidis*, *S*. *Hadar*, *S*. *Typhimurium*, *S*. *Dublin*, and *S*. *Poona*. Thermal stability test of phages showed that phages were stable at −6.5°C–50°C for 30 min, and significantly decreased (*p* < 0.05) at 60°C, and drastically decreased at 70°C. Furthermore, pH stability test of phages showed that phages were stable at pH 5-9. Phage SEpBS-1 was stable in acidic conditions. Phage titers decreased with increased salinity. The morphological characterization of the phage using transmission electron microscopy (TEM) revealed icosahedral heads and thin, long, noncontractile, flexible tails. The phage SEpBS-1 was classified as a member of the *Siphoviridae* family. The growth curve of the bacteriophage revealed that phage SEpBS-1 for SE had a latent period of 2 h, burst time of 2–3.5 h, and burst size of 166 PFU/infected cell. Phage SEpBS-1 for *S*. *Typhimurium* had a latent period of 2.5 h, burst time of 2.5–4 h, and burst size of 973 PFU/infected cell. Studying the effects of phage SEpBS-1 against *Salmonella* infection in broiler chickens found that *Salmonella* counts were slightly increased at 7 and 14 days after phage treatment. However, there was no statistically significant difference between groups (*p* > 0.05). *Salmonella* counts decreased by 40% at 14 days, while the positive control found the highest number of *Salmonella* in ceca. The application of lytic bacteriophages in the biocontrol of foodborne pathogens presents a promising approach for targeting *Salmonella*. Bacteriophage therapy offers an effective alternative to antibiotics for pathogen control.

## 1. Introduction

Antimicrobial resistance (AMR) is a critical concern in both human and animal health, particularly in food-producing animals. The AMR crisis contributes to a rising global incidence of infectious diseases in human populations, many of which have become increasingly difficult to treat with existing antimicrobial agents [1]. The forecast of the World Health Organization (WHO) in case, no action is taken to handle AMR, and the economic loss in terms of global production between now and 2050 would be US$ 100 trillion. Furthermore, the indirect costs of drug-resistant infections from morbidity, disability, premature death, and reduced effective labor supply are expected to cause a decrease in the global economic output, with estimated losses US$ 3.4 trillion annually [2]. In 2013, the Centers for Disease Control (CDC) in the USA asserted that humanity is now in the “postantibiotic” era. In May 2014, the WHO stated that the AMR crisis is becoming disastrous [3]. One of the current issues may be the results of overuse or irrational use of antibiotics in various cases, primarily in antimicrobial treatment in agricultural practices, animal healthcare, and the food-producing system [4]. Alternatives to antibiotics to treat bacterial infections are now under consideration and practices. One of the possible replacement options for antibiotics is the therapeutic use of bacteriophages [5]. Bacteriophages or phages are a group of viruses widely distributed in nature, which can infect and damage bacterial cells [6]. Phage infections have been identified across multiple bacterial species, including those from different genera such as *Salmonella* spp. and *Campylobacter* spp. Notably, phages can be isolated from the intestines of poultry, especially from free-range chickens. During processing at slaughterhouses, phages present in the gut may contaminate poultry meat, leading to their frequent presence in foods consumed by humans. In a previous study, field trials with phage application against *Campylobacter* in commercial broiler houses suggested that phages can lead to a reduction of up to log_10_ 3.29 cfu in *Campylobacter* load [7]. Phages have been revealed to reduce microorganisms on contact surfaces, carcasses, and chicken meat [8].

Controlling *Salmonella* contamination remains a significant challenge for the poultry industry. The application of lytic bacteriophages for the biocontrol of foodborne pathogens offers a promising approach to managing *Salmonella* contamination, a method deemed safe by the U.S. Food and Drug Administration [9]. The objectives of this study were to (1) isolate and (2) characterize bacteriophages to identify an effective phage for reducing *Salmonella* in the broiler gut and (3) to examine the effects of bacteriophage treatment on the gut microbiota.

## 2. Materials and Methods

### 2.1. Sample Collection

Samples of *Salmonella* phages for isolation were collected from broiler farms and poultry processing plants (20 samples), a goat farm (4 samples), and a pig farm (1 sample) in the central region of Thailand. The samples were distributed as follows: poultry processing plant environment (wastewater treatment pond and fishpond), poultry processing plants including wastewater after spraying in lairage, water from carcass washing after scalding, defeathering and offal washing, poultry farms (cloacal swab and boot swab), fecal samples from goat farm, and wastewater from pig farm. Amber bottles with a capacity of 100 mL were sterilized prior to fluid sample collection. Each bottle was filled to three-fourths of its volume with the fluid sample and securely sealed. A cloacal swab was taken by inserting a sterile swab into the cloaca. The pools of 10 swabs were placed into 0.1% (w/v) buffered peptone water (BPW). Boot swabs were performed by wearing boot swabs and walking around the poultry house. Swabs were then placed in a sterile plastic bag for transportation on ice to the laboratory. Sampling personnel wore gloves while collecting goat fecal samples, which were then placed in sterile plastic bags and transported on ice to the laboratory for analysis.

### 2.2. Preparation of *Salmonella*

To prepare *Salmonella* as a host for phage cultivation, the *Salmonella* culture on a nutrient agar (NA) slant was transferred into 10 mL of nutrient broth (NB) and incubated at 37°C for 24 h. One mL of *Salmonella* suspension was transferred to 9 mL of BPW and performed tenfold serial dilution. The number of *Salmonella* was counted on xylose lysine deoxycholate (XLD) using a spread plate with serological confirmation.

### 2.3. Isolation of Bacteriophages

The methods for phage isolation were adapted from Atterbury et al. [10]. In brief, samples were diluted to 1:9 (w/v) in NB, followed by the addition of 1.0 × 10^8^ cfu/mL of each of five *Salmonella* serovars: *Salmonella Enteritidis* (SE) ATCC DMST 15676, *S*. *Dublin* (SD), *S*. *Hadar* (SH) ATCC 10634, *S*. *Poona* (SP), and *S*. *Typhimurium* (ST) ATCC 13311 and incubated at 37°C for 24 h. A 1 mL sample from each culture was subjected to centrifugation at 4000 rpm for 5 min at 4°C, to remove the host cells. The supernatant was filtered through a 0.45-μm pore-size membrane with a plastic syringe. The agar overlay was performed by pouring the mixture of 0.8% agar, 9 mL of NB, novobiocin 50 μL at the concentrate of 1 μg/mL, and 1.0 × 10^8^ cfu/mL of each *Salmonella* serovars on NA. The 20 μL volumes of filtrate were spotted onto the surface of the upper layer of overlay and incubated at 37°C for 24 h before examination for plaques. Individual plaques were extracted from the overlay agar with a pipette and suspended in a mixture of NB 10 mL, novobiocin 10 μL, and 0.5 mL of specific *Salmonella* to phage at a concentration of 1.0 × 10^8^ cfu/mL. These suspensions were incubated at 37°C for 4 h by using a shaking incubator. Subsequently, the suspensions were centrifuged at 4000 rpm for 15 min at 4°C, and the supernatants were filtrated through 0.45-μm pore-size membranes. The filtrates were serially diluted in saline magnesium buffer (SM buffer) (50 mM Tris-HCl [pH 7.5], 0.1 M NaCl, 8 mM MgSO_4_·7H_2_O, 0.01% gelatin). A 1 mL of each dilution was poured into the mixture (0.8% agar, 10 mL of NB, 1 mL of specific *Salmonella* to phage at concentration of 1.0 × 10^8^ cfu/mL) before pouring on NA and incubated at 37°C for 24 h. The numbers of plaques were counted as plaque-forming unit (PFU) [11]. A plaque was collected and mixed with SM buffer before adding 20 μL of 2.5% (v/v) chloroform and left at room temperature for 30 min, then adding 40% glycerol (v/v) and storing at −80°C.

### 2.4. Characterization of Bacteriophages

#### 2.4.1. Determination of Host Range

Host range of bacteriophages was determined by performing spot tests [11]. Isolates of *Salmonella* spp. (SE, SD, SH, SP, and ST), *Escherichia coli*, *Pseudomonas aeruginosa*, *Micrococcus luteus*, *Staphylococcus aureus*, *Enterococcus faecalis*, *Listeria monocytogenes*, *Bacillus subtilis,* and *Pediococcus acidilactici*) each were used in this study to determine the host range of the isolated phages. Each of the bacterial isolates was transferred into NB for incubation at 37°C for 24 h. An agar overlay was performed containing a bacterial concentration of 1.0 × 10^8^ cfu/mL. The 10 μL of bacteriophage preparation (10^10^ PFU/mL) was spotted on the prepared overlay. The plates were observed for positive result by observing the appearance of lytic clear zones after incubation at 37°C for 24 h.

#### 2.4.2. Heat and pH Susceptibility Tests

For the phages capable of infecting five *Salmonella* serovars, the concentration was adjusted to 10^11^ PFU/mL. The 1 mL of phage was mixed in NB (9 mL) and measured for heat susceptibility by treating the phage stock at −6.5°C, 2°C, 6°C, 37°C, 50°C, 60°C, and 70°C for 30 min. For pH stability, the 1 mL of phage was mixed in a series of tubes containing NB (9 mL) of different pHs at 3, 5, 7, and 9 (adjusted using NaOH or HCl) and incubated at 41°C for 30 min. Bacteriophage titers were determined using the plaque assay method and repeated three times.

#### 2.4.3. Phage Stability Under Salinity Conditions

For the salinity test, the phage was adjusted to a concentration of 10^11^ PFU/mL. One milliliter of phage was added to 2% and 5% NaCl solutions and then incubated at 37°C. For the control group, 1 mL of phage was added to sterile distilled water. Bacteriophage titers were determined at 0, 2, 6, and 24 h.

#### 2.4.4. The Growth of *Salmonella* at Different Bacteriophage Concentrations

The growth of *Salmonella* at different bacteriophage concentrations to determine the multiplicity of infection (MOI) was adapted from Dallal et al. [12]. SE and ST were multiplied in NB by incubation at 37°C and centrifugation at 150 rpm for 6 h. Bacterial concentrations were adjusted to 1.5 × 10^5^ cfu/mL and then gently mixed with phages at concentration of 1.5 × 10^4^, 1.5 × 10^5^, and 1.5 × 10^6^ PFU/mL, respectively. Bacterial growth was monitored at 0, 1, 2, 3, 4, 5, 6, 7, and 8 h and compared to the control group.

#### 2.4.5. The Growth of Bacteriophage

To determine the growth of bacteriophage, a characteristic was performed according to Dallal et al. [12]. Phages were added to the NB containing SE and ST with MOI of 0.1. before incubation at 37°C and centrifugation at 100 rpm. During incubation and centrifugation, bacteriophage titers were determined using the plaque assay method every 30 min for 5.5 h. This procedure was repeated three times.

#### 2.4.6. Bacteriophage Morphological Characterization by Using Transmission Electron Microscope (TEM)

The selected phages were studied using a TEM. Bacteriophage preparation was adapted from Hayat [13]. The phage solutions at a concentration of 1.0 × 10^8^ PFU/mL were filtrated through 0.22-μm pore-size membranes for 1–3 h. The filtrates were centrifuged at 35,000 rpm for 2 h. The pellet was resuspended in phosphate buffer, and the suspension was transferred to a copper mesh grid. One drop of 2% uranyl acetate was then placed on the grid and allowed to stand for 2 min. The grid was placed onto a filter paper (Whatman No. 1) and allowed to dry for 3–5 min before observation under TEM (HT7700, Hitachi-High Tech, Tokyo, Japan) operating at 80 kV.

### 2.5. The Use of Bacteriophage in Experimental Broiler Chickens

One hundred and thirty-five broiler chicks aged one-day-old were segregated into three equal groups: The first group kept as a negative control, the second group (positive control) was infected with 500 μL (10^8^ cfu/mL) of SE per chick at one day old, while the remaining group was infected with SE at one day old and then treated with phage at 7 days old. The phage cocktails (300 μL) were given to the infected chicks (7 days of age) at a concentration of 10^11^ PFU/mL. A total of 5 birds were randomly selected from each group, at 7, 14, and 21 days of age, to collect cecum, liver, and spleen for detection of phage, and cecum, liver, and spleen were collected from the same birds for *Salmonella* count. The animal experiments were conducted in accordance with ethical guidelines and received approval from the Chulalongkorn University Animal Care and Use Committee, under approval number 2073023.

### 2.6. Microbiota Analysis

A total of three birds were randomly selected from each group at 7 and 21 days of age to collect ceca and ilea for microbiota analysis. Genomic DNA was extracted and purified using the TIANamp Stool DNA Kit (TIANGEN, China) according to the manufacturer's instructions. The sequences allowed for the amplification of the V4 region of 16S rRNA genes (adapted from [14]). The microbiota was analyzed by using next-generation sequencing (NGS) using MiSeq Reagent Kit and MiSeq System according to the manufacturer's instructions (Illumina).

### 2.7. Morphometric Analysis

At 21 days of age, three birds were randomly selected from each group, and ileal samples were collected for morphometric analysis using TEM. The villus height, villus width, and crypt depth were measured to calculate the absorption area, as adapted from [15].

### 2.8. Statistical Analysis

Results are presented as mean ± SD (standard deviation). The characterization of phage and *Salmonella* count was analyzed using one-way ANOVA with 95% confidence intervals (*p* < 0.05) followed by Tukey post hoc test to compare means.

## 3. Results

### 3.1. Isolation of Bacteriophages

*Salmonella* phages were isolated from two poultry farms, two poultry processing plants, a goat farm, and a pig farm. Of the 33 samples analyzed, 25 (75.8%) were found to contain *Salmonella* bacteriophages. A total of 63 phage isolates were identified, including 17 ST phage isolates, 12 SE phage isolates, 7 SH phage isolates, 16 SD phage isolates, and 11 SP phage isolates. The phage SEpBS-1 was selected for further testing based on its characteristics.

### 3.2. Characteristics of the Phage

#### 3.2.1. Determination of Host Range

The host ranges of the bacteriophage SEpBS-1 were determined using several types of bacteria. The bacteriophage revealed a broad host range against *Salmonella* serovars including SE, SH, ST, SD, and SP. The bacteriophage did not produce lytic plaques on *Escherichia coli*, *Pseudomonas aeruginosa*, *Bacillus subtilis*, *Listeria monocytogenes*, *Micrococcus luteus*, *Pediococcus acidilactici*, and *Staphylococcus aureus*.

#### 3.2.2. The pH Stability, Heat Stability, and Phage Stability Under Salinity Condition

The results of the pH stability, heat stability, and phage stability under salinity condition are shown in Table 1. For the pH stability, the titers of phage SEpBS-1 ranged from 5.31 ± 0.17 to 10.90 ± 0.11 log_10_ PFU/mL. The phage SEpBS-1 showed the highest titer at pH 7. The phage titers at different pH were significantly different (*p* < 0.05). The test of heat stability revealed the titers of phage SEpBS-1 ranged from 4.82 ± 0.06 to 11.52 ± 0.24 log_10_ PFU/mL. The phage SEpBS-1 showed the lowest titer at 70°C. There was no significant difference in titers between −6.5°C and 37°C (*p* > 0.05), but the higher temperature (50°C–70°C) revealed the lower titers (*p* < 0.05). For the phage stability under salinity, the titers of phage SEpBS-1 ranged from 7.84 ± 0.06 to 9.48 ± 0.00 log_10_ PFU/mL in the control group, 8.31 ± 0.03 to 9.14 ± 0.06 log_10_ PFU/mL in the 2% NaCl group, and 7.18 ± 0.18 to 9.00 ± 0.44 log_10_ PFU/mL in the 5% NaCl group. At 24 h, the titer of phage in the 5% NaCl group was significantly decreased (*p* < 0.05).

### 3.3. The Growth of *Salmonella* at Different Bacteriophage Concentrations

**Figure 1:**
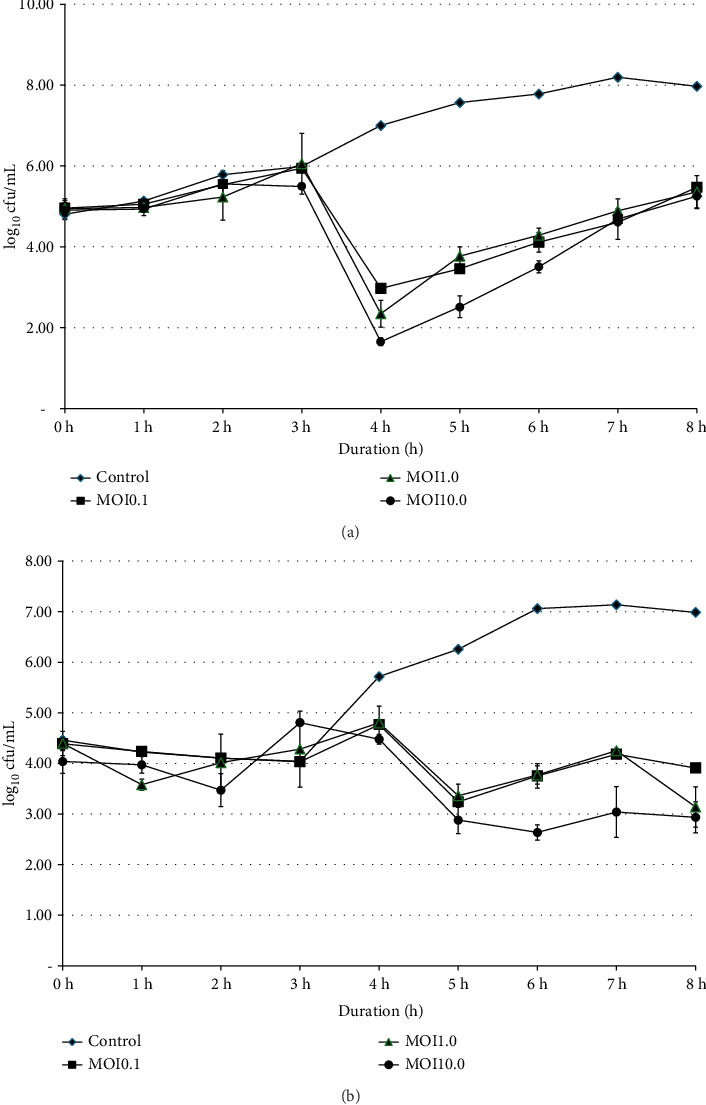
The growth of *S*. *enteritidis* (a) and *S*. *typhimurium* (b) at different phage SEpBS-1 concentrations was as follows: control (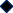), MOI of 0.1 (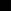), MOI of 1.0 (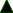), and MOI of 10 (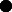).

Bacterial growths of SE and ST were monitored at 0, 1, 2, 3, 4, 5, 6, 7, and 8 h, with varying titers of phage SEpBS-1 (10^4^, 10^5^, and 10^6^ PFU/mL [MOI = 0.1, 1.0, 10]). The results of phage SEpBS-1 on SE were revealed by the bacterial counts shown in Figure 1(a). In the control group, bacterial counts had increased (3.17 log_10_ cfu/mL) after 8-h incubation. At MOI of 0.1, bacterial counts had decreased (1.98 log_10_ cfu/mL) after 4-h incubation and then increased (2.5 log_10_ cfu/mL) after 8-h incubation. At MOI of 1.0, bacterial counts decreased (2.6 log_10_ cfu/mL) after 4-h incubation and then increased (3.01 log_10_ cfu/mL) after 8-h incubation. At MOI of 10, bacterial counts decreased (3.25 log_10_ cfu/mL) after 4-h incubation and increased (3.6 log_10_ cfu/mL) after 8-h incubation.

The results of phage SEpBS-1 on ST were revealed by the bacterial counts shown in Figure 1(b). In the control group, bacterial counts increased (2.53 log_10_ cfu/mL) after 8-h incubation. At MOI of 0.1, bacterial counts decreased (1.5 log_10_ cfu/mL) after 5-h incubation and increased (0.66 log_10_ cfu/mL) after 8-h incubation. At MOI of 1.0, bacterial counts decreased (1.03 log_10_ cfu/mL) after 5-h incubation and increased (0.23 log_10_ cfu/mL) after 8-h incubation. At MOI of 10, bacterial counts decreased (1.4 log_10_ cfu/mL) after 6-h incubation and increased (0.33 log_10_ cfu/mL) after 8-h incubation.

### 3.4. The Growth of Bacteriophage

**Figure 2:**
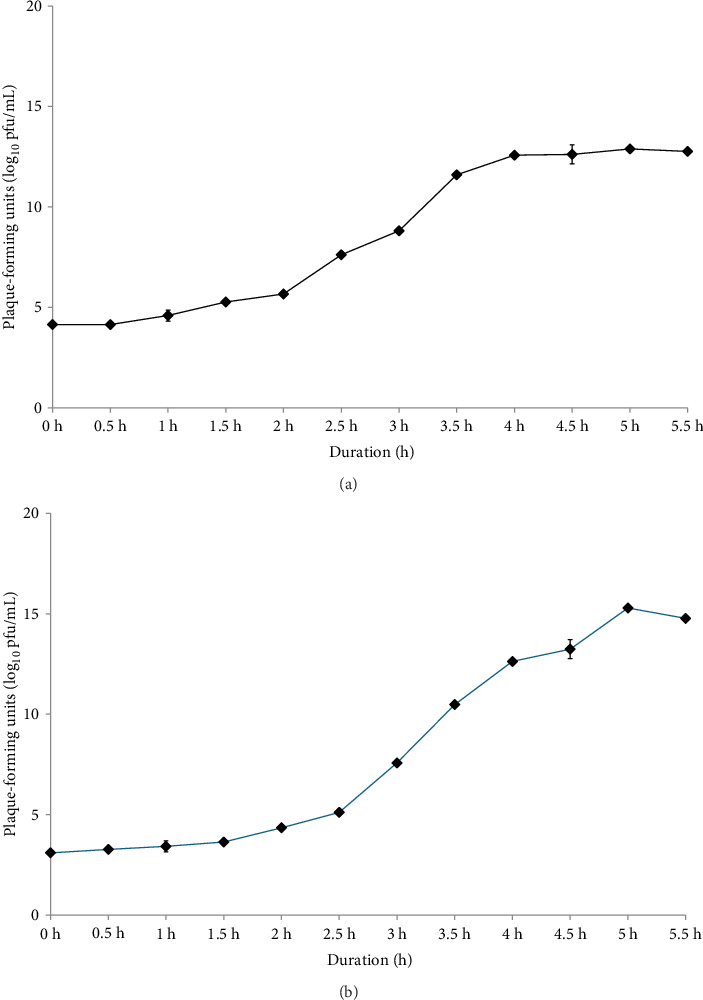
The growth of phage SEpBS-1 for S. enteritidis (a) and S. typhimurium (b).

The latent period, burst time, and burst size of the phages were determined from a one-step growth curve, as shown in Figure 2. The latent period of phage SEpBS-1 for SE was calculated as 2 h. The burst time was 2–3.5 h, and the burst size was 166 PFU/infected cell. The latent period of phage SEpBS-1 for ST was calculated as 2.5 h. The burst time was 2.5–4 h, and the burst size was 973 PFU/infected cell.

### 3.5. Bacteriophage Morphological Characterization Using TEM

**Figure 3:**
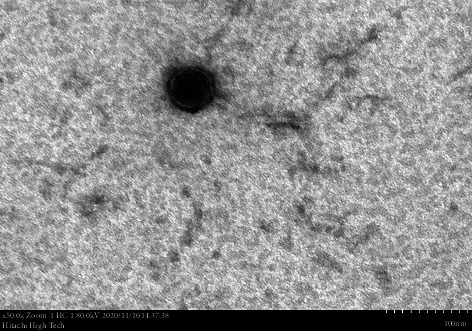
Phage SEpBS-1 under transmission electron microscope observation (50,000x).

Ultrastructural analysis of phage SEpBS-1 revealed icosahedral heads approximately 56 nm in diameter and thin, long, noncontractile, flexible tails approximately 130 nm in length, as shown in Figure 3. Based on these characteristics, phage SEpBS-1 was classified as a member of the *Siphoviridae* family.

### 3.6. *Salmonella* Counts From the Liver, Spleen, and Cecum After Treatment With Phage

*Salmonella* counts from the liver, spleen, and cecum were performed before and after treatment with phage. The results are presented in Table 2. The *Salmonella* was not detected in the negative control. At 7 days postinoculation, *Salmonella* was detected in the spleen (0.4 ± 0.89 log_10_ cfu/g) and cecum (4.5 ± 0.62 log_10_ cfu/g) of the positive control and the spleen (0.49 ± 1.10 log_10_ cfu/g) of the treatment group. At 14 days postinoculation, *Salmonella* was detected in all selected organs (0.46 ± 1.02, 0.92 ± 1.26, and 3.40 ± 3.12 log_10_ cfu/g), while the treatment group showed *Salmonella* in the liver (0.49 ± 1.12 log_10_ cfu/g) and spleen (0.52 ± 1.16 log_10_ cfu/g). At 21 days postinoculation, *Salmonella* was detected in the spleen (0.58 ± 1.29 log_10_ cfu/g) and cecum (6.13 ± 0.23 log_10_ cfu/g) of the positive control and the cecum (1.21 ± 2.27 log_10_ cfu/g) of the treatment group. The *Salmonella* counts in the cecum were significantly higher in the positive control compared to the other groups (*p* < 0.05).

### 3.7. Microbiota Analysis

#### 3.7.1. Gut Microbiota Diversity

A total of 2,502,311 sequences of the V4 region of the 16S rRNA gene were obtained from the broiler ceca at 7 and 21 days of age. Alpha diversity analysis, such as observed OTUs, Simpson, Chao 1, ACE, and PD whole tree, was used for consideration. Results of alpha diversity analysis did not show significant main effects of *Salmonella* challenge except Simpson (data not shown). At 14 days after treatment, the alpha diversity of microbiota in ceca showed no significant difference (data not shown). On the other hand, in beta diversity analysis (PCoA) (data not shown), the pattern of microbiota after *Salmonella* challenge was found that the treatment group at 7 days of age (D7Test) exhibited a tendency of separation between the positive control and the negative control. At 14 days after treatment, the treatment group at 21 days of age (D21Test) was tended to separate from the negative (D21Neg) and positive control (D21Pos) groups.

#### 3.7.2. Diversity and Community Structure of Gut Microbiota During Chicken Development

**Figure 4:**
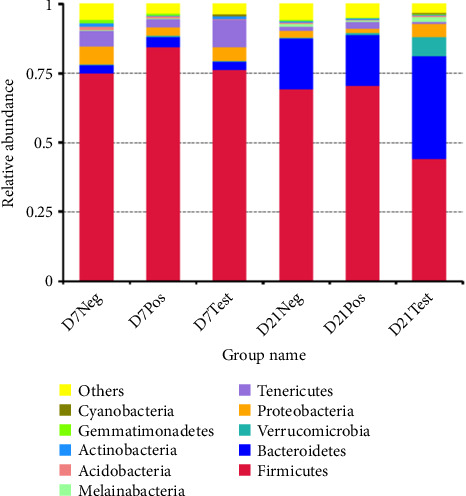
The relative abundances of the cecal microbiota at the phylum level. Abbreviations: D7Neg, negative control at 7 days of age; D7Pos, positive control at 7 days of age; D7Test, treatment group at 7 days of age; D21Neg, negative control at 21 days of age; D21Pos, positive control at 21 days of age; D21Test, treatment group at 21 days of age.

**Figure 5:**
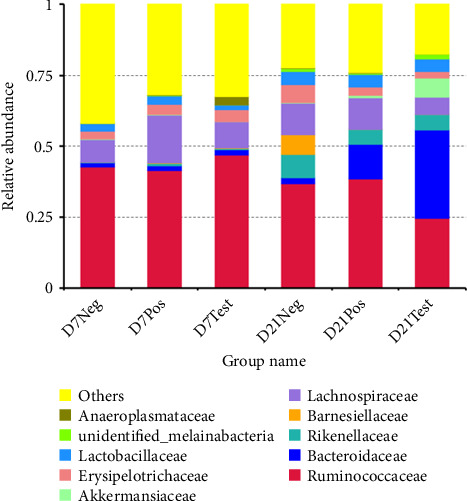
The relative abundances of the cecal microbiota at the genus level. Abbreviations: D7Neg, negative control at 7 days of age; D7Pos, positive control at 7 days of age; D7Test, treatment group at 7 days of age; D21Neg, negative control at 21 days of age; D21Pos, positive control at 21 days of age; D21Test, treatment group at 21 days of age.

At the phylum level, the bacterial sequences from the treatment group at 21 days of age were composed predominantly of the phyla *Firmicutes*, *Bacteroidetes*, *Verrucomicrobia*, and *Proteobacteria*, and this group showed higher proportions of *Bacteroidetes* and *Verrucomicrobia*, compared to the other groups, as depicted in Figure 4. At the family level, the treatment group at 21 days of age consisted largely of *Bacteroidaceae* and *Ruminococcaceae*. *Bacteroidaceae* was the highest proportion. The proportion of *Akkermansiaceae* in this group was higher when compared to the other groups. At the genus level, gut microbiota of this group was composed of *Bacteroides*, *Alistipes*, unidentified *Ruminococcaceae*, *Barnesiella*, *Akkermansia*, *Erysipelatoclostridium*, *Subdoligranulum*, *Lactobacillus*, *Faecalibacterium*, and unidentified *Lachnospiraceae*. A higher abundance of *Bacteroides* and *Akkermansia* was observed when compared to other groups (Figure 5).

### 3.8. Morphometric Analysis

The villus height, villus width, and crypt depth of the ileum were measured to calculate the absorption area (Table 3). Villus surface area was significantly higher in the treatment group when compared to other groups (*p* < 0.05).

## 4. Discussions

*S*. *enterica* is classified into over 2600 serovars, which can cause disease in humans and different kinds of animals [16]. Additionally, *S*. *enterica* is known to exhibit resistance to a wide range of antibiotics [17, 18], including those from higher generations of antibiotic applications [19]. Contamination by *Salmonella* is a common issue in poultry meat processing. This microorganism can increase meat products during transportation and distribution, especially international transportation. Therefore, it is necessary to prevent and reduce microbial contamination before packaging, during transportation and distribution. The lytic bacteriophages are a safe and effective choice for control of *Salmonella* [9].

In this experiment, bacteriophages were isolated using the enrichment method, and one of the bacteriophages exhibited high lytic efficiency against various *Salmonella* isolates. The bacteriophages were named based on their specific hosts and the locations of their discovery, following the naming convention outlined by Jiang et al. [20]. One of the isolated bacteriophages, with suitable properties, was selected and named SEpBS-1 for further study.

Bacteriophages will target and kill specific strains of bacteria. They cannot replicate without the bacterial cell [21]. The characterization results showed that the phages are highly specific to SE, SH, ST, SD, and SP. The thermal stability test of phages showed that phages were stable at −6.5°C–50°C for 30 min, significantly decreased (*p* < 0.05) at 60°C, and maximally decreased at 70°C. The pH stability test showed that phages were stable at a pH of 5–9. Phage SEpBS-1 was more stable in the acidic solution than alkaline solution, according to Rahaman et al. [11]. The *Salmonella* phages which were isolated from a poultry farm environment were stable at 50°C and 60°C for 1 h. They survived at pH 4–9, but their titers decreased at pH 2–3. NaCl stability showed individual differences for individual phages in a study of NaCl tolerance of staphylococcal phages [22]. In this study, titers of phage SEpBS-1 decreased in response to increased salinity. The morphological characterization of phage SEpBS-1 using TEM revealed icosahedral heads with a diameter of 56 nm and thin, long, noncontractile, flexible tails measuring 130 nm in length. Based on these characteristics, phage SEpBS-1 was classified as a member of the *Siphoviridae* family.

The growth of *Salmonella* at different bacteriophage concentrations revealed that SEpBS-1 significantly decreased bacterial counts after 4 h incubation at all MOI on SE. SE counts were lowest at MOI of 10. The bacterial counts varied directly with time at every MOI. For phage SEpBS-1 on ST, bacterial counts were significantly decreased after 5-h incubation at all MOI and tended to increase with time except for MOI of 10, for which ST counts were 2.63–3.03 log_10_ PFU/mL. According to the one-step growth curve, phage SEpBS-1 on SE and ST had a latent period of 2 and 2.5 h, respectively. A previous study reported that some *Salmonella* phages had long latent periods [23]. The burst time of phage SEpBS-1 was 2–3.5 h. The phage SEpBS-1 had a shorter latent period and burst time on SE than on ST, but had a smaller burst size on SE than on ST. Both latent period and burst size depend on the type of bacteriophage [24], the host cell, culture media, pH, and temperature [25].

The test of the effect of phage SEpBS-1 against *Salmonella* infection in broilers found that *Salmonella* counts were slightly increased at 7 and 14 days after phage treatment. There was no statistically significant difference between groups (*p* > 0.05). *Salmonella* counts decreased by 40% at 14 days after phage treatment, while the positive control had the highest number of *Salmonella* in ceca. Bacteriophages may be efficient for *Salmonella* control. However, as chickens age, their immunity could respond to eliminate *Salmonella*. Adhikari and colleagues [26] studied the efficiency of bacteriophages against SE. The 0.2% of bacteriophages significantly reduced numbers of SE in the ceca when compared to the positive control. Furthermore, an increased expression of proinflammatory cytokine and chemokine genes was reported to be associated with increased resistance to SE, including higher levels of IL-1B, IL-6, IL-8, IL-18, and chemokine ligand-2 (CCLi2) in heterophils, monocyte-derived macrophages, the ceca, and cecal tonsil [27, 28]. The effect of a phage preparation can be mediated through two primary mechanisms. The first mechanism involves the direct killing of bacterial cells by bacteriophage virions during the lytic cycle. The second mechanism relies on inducing an immune response, either by the phage particles themselves or by other components of the phage preparation [29].

The morphology of the intestines reflects the health status of the poultry gut and its immune function. According to Sarrami et al. [30], the rapid development of intestinal integrity provides a suitable environment for bacteria to colonize and thrive. In our experiment, the treatment group showed significantly better parameters compared to the positive control group (*p* < 0.05), suggesting that phages can promote intestinal maturation. The ecology of the diverse microbiota in the poultry gut is closely linked to the stability of the gut microbiota. Bacteriophages can alter gut bacterial communities. Sequencing the V4 region of the 16S RNA characterized the bacterial communities in each broiler cecum. Beta diversity analysis (PCoA) can assess microbiota patterns. After treatment, the treatment group at 21 days of age (D21Test) showed a tendency to separate from both the negative (D21Neg) and positive control (D21Pos) groups. Alpha diversity analysis was used to characterize the bacterial communities. In line with the findings of Chang et al. [31], the *Salmonella* challenge in day-old chicks did not result in significant alterations to the bacterial communities. However, the Simpson index revealed a significant difference (*p*=0.049), indicating a shift in community diversity.

The microbiota of broiler chickens has been estimated to surpass 900 bacterial species. The variation in microbiota composition may be explained by different host characteristics and environmental factors [31, 32]. In this study, *Salmonella* inoculation and bacteriophage treatment decreased the abundance of *Firmicutes*, while the abundance of *Bacteroidetes*, *Verrucomicrobia*, and *Proteobacteria* was increased.

The most pathogenic bacterial species, including *Salmonella*, *Escherichia coli*, and *Shigella*, are members of the phylum *Proteobacteria*. These bacteria have the ability to attach, colonize, and invade the intestinal epithelium, leading to disease in humans and animals. The increase of phylum *Bacteroidetes* and *Verrucomicrobia* may indicate a healthy gut in poultry. Carbohydrates are efficiently digested by members of the phylum *Bacteroidetes* [33]. Previous studies described substantially different *Bacteroides* population levels (10^7^ to 10^10^ cfu/g) in ceca or feces of chickens at different ages (14–40 days of life) [34]. *Bacteroides thetaiotaomicron*, which is one of the most excellent fiber-digesting bacteria, is a member of the phylum *Bacteroidetes*. In this study, the highest average abundance of phylum *Bacteroidetes* was 226.33 and was significantly different (*p* < 0.05) among the groups. *B*. *thetaiotaomicron* can degrade dietary fiber by releasing outer membrane vesicles (OMVs) containing glucohydrolase. A previous study showed that *B*. *thetaiotaomicron* can ferment cello-polysaccharides to produce short-chain fatty acids (SCFAs) to provide energy for the host [35]. The highest average abundance of *Akkermansia muciniphila* (phylum *Verrucomicrobia*) was 7428.67, and abundances differed significantly (*p* < 0.05). *A*. *muciniphila* improves intestinal immunity and affects glucose metabolism and lipid metabolism which can suppress obesity [36]. Zhu et al. [37] found that *A*. *muciniphila* colonized in the intestine and then relieved intestinal mucosal damage in chicks caused by *S*. *pullorum*.

The results of this study on the efficiency of phage SEpBS-1 reinforce the concept of using bacteriophages as a biological control for *Salmonella* in the poultry industry. Phage SEpBS-1 effectively reduced *Salmonella* in the organs of birds 14 days after treatment. However, *Salmonella* was most frequently detected in the cecum of the treatment group. The use of bacteriophages provides a promising alternative to antibiotics. The application of lytic bacteriophages in the biological control of foodborne pathogens is an effective strategy for controlling *Salmonella*. The U.S. Food and Drug Administration has approved the use of bacteriophages on meat products as safe [9].

## 5. Conclusion

The findings of this study highlight the viability of bacteriophage SEpBS-1 as a potential antibiotic alternative for managing *Salmonella* in broiler chickens. Isolated from various animal farms and processing plants in Thailand, SEpBS-1 was able to infect multiple *Salmonella* serovars, indicating broad host specificity and robustness under various environmental conditions, including temperature and pH ranges commonly found in poultry environments. While in vivo tests showed a modest reduction in *Salmonella* counts in treated broilers, these reductions were not statistically significant, suggesting that while phage therapy is promising, further optimization in phage dosage, application frequency, or delivery methods may be necessary to achieve more pronounced results.

Overall, the findings underscore the potential of lytic bacteriophages as a biocontrol measure for *Salmonella*, especially as the poultry industry moves toward reducing antibiotic usage. Continued investigation into the impact of phage therapy on gut microbiota and resistance development will be key to establishing bacteriophages as a sustainable and effective approach in poultry production.

## Figures and Tables

**Table 1:** Stability of phage SepBS-1 under various conditions.

Parameters	Levels tested	Contact time (h)	Mean ± SD (log10 PFU/mL)
pH	3	0.5	5.31 ± 0.17^d^
	5	0.5	10.46 ± 0.10^b^
	7	0.5	10.90 ± 0.11^a^
	9	0.5	8.97 ± 0.07^c^

Temperature (°C)	−6.5	0.5	11.07 ± 0.23^a^
	2	0.5	11.52 ± 0.24^a^
	37	0.5	11.46 ± 0.10^a^
	50	0.5	9.72 ± 0.06^b^
	60	0.5	9.03 ± 0.27^b^
	70	0.5	4.82 ± 0.06^c^

Salinity	0%	0	9.48 ± 0.00
		2	8.87 ± 0.03
		6	8.16 ± 0.01
		24	7.84 ± 0.06
	2%	0	9.14 ± 0.06
		2	8.75 ± 0.07
		6	8.41 ± 0.37
		24	8.31 ± 0.03
	5%	0	9.00 ± 0.44^a^
		2	8.62 ± 0.31^a^
		6	8.78 ± 0.11^a^
		24	7.18 ± 0.18^b^

*Note:* Different superscripts in the same row mean statistically significant (*p* < 0.05).

**Table 2:** *Salmonella* counts (means ± standard error) from the liver, spleen, and cecum after treatment with phage (*n* = 5).

Groups	Days postinoculation of *Salmonella* enteritidis		
	Day 7	Day 14	Day 21
Negative control			
*Salmonella* counts (log_10_ cfu/g)			
Liver	ND	ND	ND
Spleen	ND	ND	ND
Cecum	ND	ND	ND
%Positive	0	0	0
Positive control			
*Salmonella* counts (log_10_ cfu/g)			
Liver	ND	0.46 ± 1.02^A^	ND
Spleen	0.4 ± 0.89^a,B^	0.92 ± 1.26^a,A^	0.58 ± 1.29^a,B^
Cecum	4.5 ± 0.62^b,A^	3.40 ± 3.12^b,A^	6.13 ± 0.23^a,A^
%Positive	80	100	80
Treatment			
*Salmonella* counts (log_10_ cfu/g)			
Liver	ND	0.49 ± 1.12^A^	ND
Spleen	0.49 ± 1.10^a,B^	0.52 ± 1.16^a,A^	ND
Cecum	ND	ND	1.21 ± 2.27^B^
%Positive	80	100	40

*Note:* ND = not detected by either direct plating or enrichment for *Salmonella*.

^a-b^Values within a row with different superscripts are significantly different (*p* < 0.05).

^A-B^Values within a column with different superscripts are significantly different (*p* < 0.05).

**Table 3:** The values of evaluated parameters of ileal, villi, and villus surface area (*n* = 3 in each group) at 21 days old.

Parameter	Groups of experiment		
	Negative control	Positive control	Treatment
Ileal villus height (μm)	183.33 ± 25.00^b^	152.77 ± 63.05^b^	327.77 ± 107.85^a^
Base width of the ileal villus (μm)	40.00 ± 7.07^a^	27.78 ± 4.41^b^	37.78 ± 6.67^a^
Ileal crypt depth (μm)	27.78 ± 4.01^a^	14.44 ± 7.26^b^	36.67 ± 10.00^a^
Villus surface area (mm^2^)	0.024 ± 0.010^b^	0.013 ± 0.005^b^	0.040 ± 0.013^a^

*Note:* Values are means ± standard error. Mean values with different letters in the same row differ significantly (*p* < 0.05).

## Data Availability

Data will be made available on reasonable requests.
